# 678. Standardizing Nursing Orientation with Burn Therapy

**DOI:** 10.1093/jbcr/irag033.559

**Published:** 2026-04-06

**Authors:** Renée Warthman, Stacey Richerbach, Derek O Murray, Emily McAuliff, Gabriella Viera, Karen J Richey, Kevin N Foster

**Affiliations:** Diane & Bruce Halle Arizona Burn Center - Valleywise Health, Phoenix, AZ; Diane & Bruce Halle Arizona Burn Center - Valleywise Health, Phoenix, AZ; Diane & Bruce Halle Arizona Burn Center - Valleywise Health, Phoenix, AZ; Diane & Bruce Halle Arizona Burn Center - Valleywise Health, Phoenix, AZ; Diane & Bruce Halle Arizona Burn Center - Valleywise Health, Phoenix, AZ; Diane & Bruce Halle Arizona Burn Center - Valleywise Health, Phoenix, AZ; Diane & Bruce Halle Arizona Burn Center - Valleywise Health, Phoenix, AZ

## Abstract

**Introduction:**

Management of burn-injured patients relies on strong multidisciplinary teamwork to optimize patient care and outcomes. At our center, an eight-year retrospective review revealed RN turnover rates exceeding both hospital-wide and national burn-specific rates. Combined with orientee feedback, these findings highlighted the need for a more structured interprofessional approach RN orientation. Previously, the new RN orientation included a shadow shift with a burn therapist; however, the experience varied widely for RNs, risking underemphasis of rehabilitation during acute burn training. To address this gap, we sought to standardize burn therapy content within RN orientation.

**Methods:**

A collaborative effort between burn-certified therapists and RNs took place to define an optimized approach to the new hire RN orientation with burn therapy. Through this process, an orientation list of skills and topics was developed. The ABA burn nurse competencies were used to inform the selection of skills and topics upon which to base orientation.

**Results:**

The checklist was tailored to the setting in which the RN would be working and was formatted to include the method of evaluation. The inpatient checklist consisted of 50 items, and the outpatient checklist consisted of 37 items. Competencies include verbalizing the role of therapy, purpose of common therapy interventions, and logistics (gym location, therapy office, locating a patient’s therapist, orthotic/positioning orders in the EMR), and demonstrating therapy-related skills pertinent to nursing (residual limb wrapping, ace wrap, application of common orthoses, functional hand dressings). The ABA burn nurse competencies related to rehabilitation (7.1, 7.2, 7.3) were also included.

Since implementation, 80% of newly onboarded RNs (n = 24) and 18.9% of RN core staff have completed the standardized therapy orientation. Importantly, this process facilitated 100% coverage of the Burn Nurse Competency Initiative during orientation.

**Conclusions:**

Utilization of a competency-based, burn expert-informed checklist provided a framework for consistent, interdisciplinary RN education and established an agreed upon set of rehabilitation competencies within our burn center. Ongoing monitoring is being conducted to identify additional opportunities for training enhancement.

**Applicability of Research to Practice:**

This model of multidisciplinary education and collaboration is scalable and adaptable to resource-limited centers. It may be used to guide RN orientations to better facilitate understanding of therapies’ role in burn care, enhance RN competency and satisfaction, and improve care quality. Lastly, there is potential for this checklist to support burn nurse certification preparation, making it not only orientation-focused but also career-advancing.

**Funding for the study:**

N/A.

